# Ectopic expression of mutated type 2C protein phosphatase *OsABI-LIKE2* decreases abscisic acid sensitivity in *Arabidopsis* and rice

**DOI:** 10.1038/s41598-018-30866-z

**Published:** 2018-08-17

**Authors:** Akira Endo, Chika Egawa, Mihoko Oohashi, Ayano Meguro-Maoka, Etsuo Shimosaka, Yutaka Sato

**Affiliations:** 1 Hokkaido Agricultural Research Center, National Agriculture and Food Research Organization (NARO), Toyohira, Sapporo, 062–8555 Japan; 2 Division of Bio-systems Sustainability, Graduate School of Agriculture, Hokkaido University, N9 E9, Kita-ku, Sapporo, 060-8589 Japan; 3 Present Address: Plant Genome Engineering Research Unit, Institute of Agrobiological Sciences, NARO, 2-1-2 Kannondai, Tsukuba, 305-8602 Japan; 4 Present Address: Institute for Agro-Environmental Sciences, NARO, 3-1-3 Kannondai, Tsukuba, 305-8604 Japan; 5 Present Address: Biomedical research, Education instrumentation center, Sapporo Medical University, S1 W17, Chuo-ku, Sapporo, 060-8556 Japan

## Abstract

Abscisic acid (ABA) is a phytohormone that is necessary for stress adaptation. Recent studies have reported that attenuated levels of ABA improved grain yield and seedling growth under low temperature in cereals. To improve plant growth under low temperature, we attempted to generate ABA-insensitive transgenic rice by expressing a clade A type 2C protein phosphatase (OsPP2C), OsABIL2, with or without the mutation equivalent to the *Arabidopsis abi1-1* mutation. A yeast two-hybrid assay revealed that the interaction between OsABIL2 and a putative rice ABA receptor, OsPYL1, was ABA-dependent, and the interaction was lost with amino acid substitution from glycine to aspartic acid at the 183rd amino acid of the OsABIL2 protein, corresponding to *abi1-1* mutation. The constitutive expression of *OsABIL2* or *OsABIL2*^*G183D*^ in *Arabidopsis* or rice decreased ABA sensitivity to differing degrees. Moreover, the transgenic rice expressing *OsABIL2*^*G183D*^ exhibited improved seedling growth under low temperature, although the transgenic lines showed unfavorable traits, such as viviparous germination and elongated internodes. These results indicated that the introduction of *abi1-1* type dominant mutation was also effective in OsABIL2 at decreasing ABA sensitivity in plants, and the attenuation of ABA sensitivity could be an alternative parameter to improve rice performance under low temperatures.

## Introduction

Abscisic acid (ABA) is a phytohormone that controls various physiological processes such as seed maturation, germination, guard cell closure and adaptations to biotic or abiotic stresses. The levels of cellular ABA are varied according to physiological processes, and are controlled by the balance among biosynthesis, catabolism and transport^1,2^. Increased ABA activates signal transduction, and triggers a substantial number of gene expressions or protein interactions, to adapt to biotic or abiotic stresses^3–5^.

ABA signal transduction is initiated by the perception of ABA by the authentic ABA receptor, PYR/PYL/RCAR^6,7^. The identification of a cytosolic ABA receptor (PYR/PYL/RCAR) revealed the core component of ABA signaling in *Arabidopsis*^8,9^. The core ABA signaling component consists of three different proteins, including PYR/PYL/RCAR, clade A type 2C protein phosphatase (PP2C), and SNF1-related protein kinases 2 (SnRK2)^10^. When the level of endogenous ABA is low, PP2C interacts with SnRK2 via the catalytic cleft of each protein to inactivate SnRK2^11^. In the presence of high levels of ABA, PYR/PYL/RCAR binds to ABA and inhibits the activity of PP2Cs by docking to the catalytic cleft of PP2C^11^. The ABA dependent interaction between ABA receptors and PP2Cs restores the activity of SnRK2, resulting in the activation of ABA signaling by phosphorylating transcriptional factors, kinases, or phosphatases^5,12^. The core ABA signaling component seems to be conserved in higher plants since the genes encoding its core component were widely observed in known plant genomes, and have been isolated from various crops such as soybean, *Brachypodium*, tomato, and rice^13–15^.

ABA possesses two opposite effects related to stress adaptation in plants. On the one hand, genetic enhancements of ABA metabolism or sensitivity effectively improve drought stress tolerance in various plant species at the vegetative stage^16–18^. In addition, the exogenous application of ABA itself or chemical agents affecting ABA metabolism or signaling also enhances stress tolerance^18,19^. Chemical screening of ABA agonists revealed that quinabactin selectively binds to dimeric ABA receptors, and that exogenous application of the chemical is enough to activate the ABA response to drought stress at the vegetative stage in *Arabidopsis* and in crops including soy bean, barley and maize^18^.

On the other hand, when crops were subjected at the reproductive stage to environmental stresses such as high temperatures, drought, or cold, stress-induced pollen sterility led to the loss of grain or fruit. This phenomenon has been very problematic for agricultural crop production. Ji *et al*.^20^ found that drought-induced pollen sterility was closely related to the ABA level. The ABA level in the spikes of drought resistant varieties was lower than in those of drought sensitive varieties. Additionally, the pollen specific expression of the ABA inactivating enzyme, ABA 8′-hydroxylase, alleviated cold stress-induced pollen sterility in rice^20^. Therefore, the signal or level of ABA in plants needs to be controlled in accordance with the developmental stage or tissue under various stress conditions to enhance stress resistance via the manipulation of ABA metabolism or signal transduction.

Our group previously reported that decreased ABA levels caused by the overexpression of ABA 8′-hydroxylase improved seedling vigor under low temperature conditions^21^. The transgenic rice grew faster than wild-type rice under low temperature conditions, although the lower ABA level in the transgenic rice caused the ‘wilty phenotype’ reported as the typical phenotype of ABA-deficient mutants^21^. In line with previous reports, we hypothesized that decreased ABA sensitivity could improve plant growth under low temperature conditions. To decrease the ABA sensitivity of rice, we utilized mutated PP2C found in *abi1-1*, *Arabidopsis* ABA insensitive mutant. The *abi1-1* mutant showed obvious ABA insensitivity, such as a wilty phenotype caused by excess transpiration from ABA insensitive guard cells in addition to ABA resistant germination^22,23^. The mutation was found in PP2C and led to amino acid substitution from glycine to aspartic acid at 180th amino acid in ABI1^22,23^. Revertant screening of *abi1-1* revealed that most of the mutations were intragenic mutations in *abi1-1* gene, indicating that ABI1 acts as negative regulator of ABA signaling and *abi1-1* type mutation caused dominant negative mutations by amino acid substitution^24^. Identification of PYR/PYL/RCAR showed that the abi1-1 protein did not interact with ABA receptors with or without ABA^8^. Crystal structure analysis of the complex consisting of ABA receptors and HAB1 revealed that *abi1-1* type mutation inhibited the interaction between ABA receptors and HAB1^25^. In addition, rational structure analysis of the complex predicted that amino acid substitution from tryptophan (W) to alanine (A) at the 300^th^ amino acid in HAB1 would inhibit the interaction of HAB1 with ABA receptors^25^. HAB1^W300A^ does not interact with ABA receptor in yeast, and the ectopic expression of HAB1^W300A^ in *Arabidopsis* presented ABA-resistant germination and wilty phenotypes as a result of ABA insensitivity, indicating that ABA-receptor-insensitive PP2C could reduce ABA sensitivity in plants^25^. Several studies have reported that the ectopic expression of clade A PP2Cs harboring the *abi1-1* type dominant mutation showed a stronger ABA insensitivity than that of regular clade A PP2C in dicotyledonous plants such as *Arabidopsis* and poplar^26,27^. There are no reports of ABA-insensitive transgenic monocotyledonous plants being generated that express mutated PP2C. In the present study, we tried to express mutated *OsABIL2* as well as regular *OsABIL2* in *Arabidopsis* and rice to examine the effects of the *abi1-1* type mutation in OsABIL2 on the ABA sensitivity and agricultural traits of rice. Moreover, we investigated whether transgenic rice with reduced ABA sensitivity presented improved growth under low temperature conditions or not.

## Results

### OsABIL2 interacted with OsPYL1 in an ABA-dependent manner in yeast

**Figure 1:**
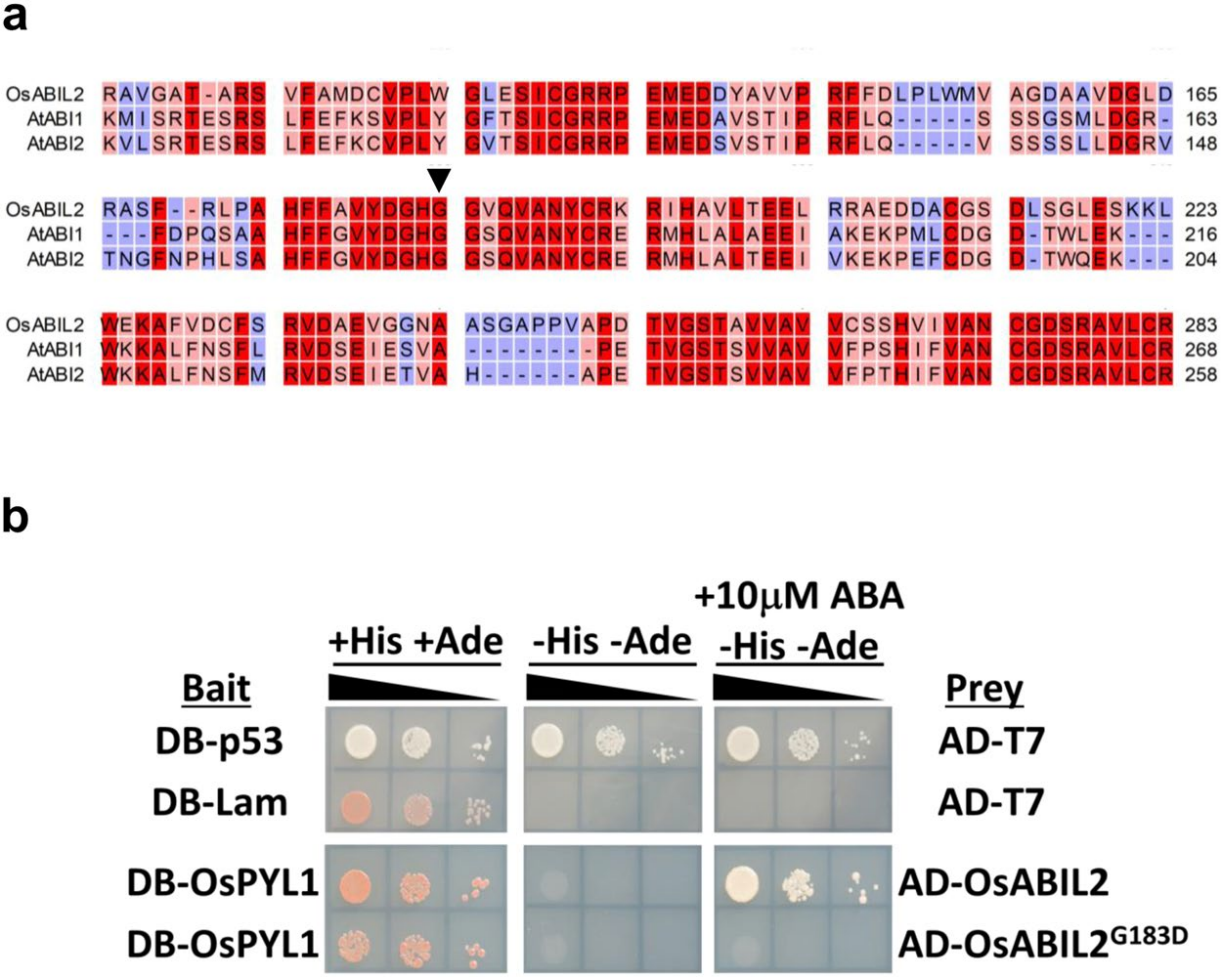
Sequence alignment to identify 183rd glycine in OsABIL2 and yeast two-hybrid assay to confirm the interaction of the OsABIL2 protein with OsPYL1. (**a**) Amino acid sequence alignment of OsABIL2, ABI1, and ABI2 to reveal the position of glycine in OsABIL2 corresponding to the 180th glycine in ABI1. The partial sequence alignment was presented. The position of the 180th glycine in ABI1 is indicated by a black arrow head. Right side numbers show the positions of amino acid residue in each protein sequence. Dashes indicate gaps in the sequence alignment results. The red color indicated that three residues were identical. The pink color presented by two of the three residues are identical and the blue color represented no matching. (**b**) The effect of G183D mutation in OsABIL2 on the interaction between OsABIL2 and OsPYL1. The interaction was examined by the GAL4-based yeast two-hybrid assay on medium lacking histidine (His) and adenine (Ade) in the presence or absence of 10 μM ABA. The black slope indicated the dilution series of yeast cells. BD presented the Gal4-DNA-binding domain, and AD indicated the Gal4-activation domain. The pair of DB-p53 and AD-T7 is a positive control, indicating the interaction between the p53 and T7 proteins. The pair of DB-Lam and AD-T7 is a negative control, showing the absence of an interaction between Lam and T7.

We initially cloned *OsABIL2* because the OsABIL2 protein has high similarity to ABI1 and was also shown to be involved in ABA signaling in rice^28^. PP2Cs participated in ABA signaling are known to interact with ABA receptors in an ABA-dependent or -independent manner. *OsPYL1* was isolated and characterized as an ortholog of AtPYR1 in rice^29^. AtPYR1 is a member of the dimeric ABA receptors that interact with clade A PP2C in an ABA-dependent manner^30^. Previous papers have reported that the ectopic expression of abi1-1 mutated protein in plants obviously caused ABA insensitive phenotypes^26^. The mutation of *abi1-1* resulted in substitution of the 180th amino acid from glycine to aspartic acid in *Arabidopsis* ABI1 protein and homogenous amino acid substitution in *ABI2* and *HAB1* also led to ABA insensitivity^27,31,32^. We first investigated the position of glycine in OsABIL2 corresponding to the 180th glycine of ABI1. Sequence alignment of ABI1, ABI2, and OsABIL2 proteins revealed that 180th glycine of ABI1 corresponded to the 183rd glycine of OsABIL2 (Fig. 1a). To examine the effect of *abi1-1* type mutation in OsABIL2 on the interaction between OsABIL2 and OsPYL1, a yeast two-hybrid assay was performed. As shown in Fig. 1b, OsABIL2 interacted with OsPYL1 in an ABA-dependent manner. The interaction between OsABIL2 and OsPYL1 was abolished by the amino acid substitution from glycine to aspartic acid at the 183rd amino acid of the OsABIL2 protein (Fig. 1b). The wild-type and mutant-type OsABIL2 genes were designated OsABIL2^WT^ and OsABIL2^G183D^, respectively. It was highly possible that the ectopic expression of *OsABIL2*^*G183D*^ in plants could decrease ABA sensitivity.

### Constitutive expression of *OsABIL2*^*G183D*^ caused ABA insensitivity during germination to seedling establishment, and excess water loss of detached shoots in *Arabidopsis*

To investigate the *in vivo* function of OsABIL2, we created transgenic *Arabidopsis* lines. This aimed to examine whether the constitutive expression of *OsABIL2*^*WT*^ or *OsABIL2*^*G183D*^ would reduce ABA sensitivity in *Arabidopsis* or not.

**Figure 2:**
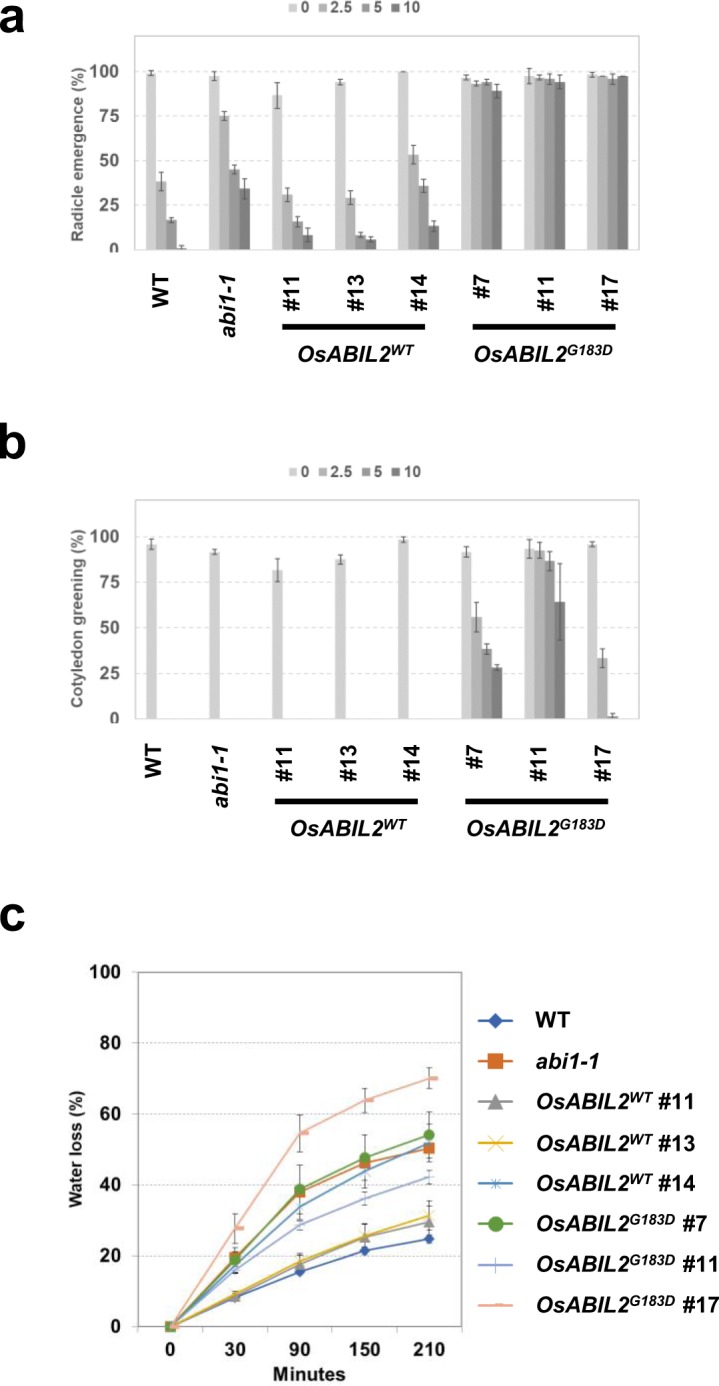
The constitutive expression of *OsABIL2*^*WT*^ and *OsABIL2*^*G183D*^ in *Arabidopsis*. (**a**,**b**) The effects of ABA on radicle emergence and cotyledon greening. In all experiments, 40 seeds of each line were used, and each experiment was performed in triplicate. Values with standard deviations (SD) are represented (n = 3). Both rates were values at four days after stratification treatment. (**c**) Water loss assay of *OsABIL2*^*WT*^ and *OsABIL2*^*G183D*^ lines. Shoots from each genotype were detached from four-week-old plants, and their initial weights were recorded before starting the water loss assay. Weights were measured at 30, 90, 150, and 210 min after detachment. Bars indicate SD (n = 5). The experiment was repeated twice.

Seed germination and seedling establishment are sequential processes that are arrested by the exogenous application of ABA^33,34^. *Arabidopsis* ABA-insensitive mutants could germinate and set green cotyledon on ABA-supplemented media^35^. To examine the ABA sensitivity of the transgenic lines that expressed *OsABIL2*^*WT*^ or *OsABIL2*^*G183D*^ during seed germination, the seed germinability of transgenic *Arabidopsis* was compared to those of the wild-type (WT) or *abi1-1* mutant on media containing 0, 2.5, 5, and 10 μM of ABA. Radicle emergence from the seeds was applied as a criterion to evaluate seed germination. There were no differences observed in the germination rates among all genotypes on the medium without ABA (Fig. 2a). The germination rates of *OsABIL2*^*WT*^ lines were higher than those of WT, and lower than those of the *abi1-1* mutant, on media containing ABA (Fig. 2a). The germination rates of *OsABIL2*^*G183D*^ transgenic lines were more than 90% on the media containing ABA. The ABA sensitivity of *OsABIL2*^*G183D*^ lines was lower than that of the *abi1-1* mutant.

The rate of seedling establishment was measured by counting the rate of cotyledon greening on ABA-containing media. The rates of cotyledon greening were comparable across all genotypes in the absence of ABA (Fig. 2b). WT, *abi1-1*, and *OsABIL2*^*WT*^ transgenic lines did not show cotyledon greening on media containing ABA (Fig. 2b). Transgenic lines expressing *OsABIL2*^*G183D*^ presented cotyledon greening, although the rates of cotyledon greening were different among *OsPP2C*^*G183D*^ lines (Fig. 2b).

ABA insensitive mutants exhibit the wilty phenotype since the inability to close stomata causes excess transpiration from leaves^25,26^. To examine the wilty phenotype of the *OsABIL2*^*WT*^ and *OsABIL2*^*G183D*^ lines, a water loss assay was performed by measuring the weight of detached shoots from all genotypes at regular time intervals (Fig. 2c). As shown in Fig. 2c, the water loss rates observed in the *OsABIL2*^*WT*^ transgenic lines were higher than that of WT, and lower than that of *abi1-1*. Although line #17-6 of *OsABIL2*^*G183D*^ showed the highest water loss rate among all genotypes, the other two lines of *OsABIL2*^*G183D*^ similarly lost fresh weight in a manner that was similar to that of *abi1-1* (Fig. 2c). From these results, the ABA sensitivity of *OsABIL2*^*WT*^ lines was shown to be intermediate between that of the WT and that of the *abi1-1* mutant, and *OsABIL2*^*G183D*^ lines were shown to be more insensitive to ABA than the *abi1-1* mutant. These results clearly indicated that *OsABIL2*^*WT*^ could have a function in *Arabidopsis* ABA signaling, and the ectopic expression of *OsABIL2*^*G183D*^, but not *OsABIL2*, resulting in a strong ABA insensitivity in *Arabidopsis*.

### Ectopic expression of *OsABIL2*^*G183D*^ reduced the ABA sensitivity in rice seed

To examine whether or not the constitutive expression of *OsABIL2*^*WT*^ or *OsABIL2*^*G183D*^ in rice could reduce the ABA sensitivity, we generated transgenic rice harboring *GUS*, *OsABIL2*^*WT*^, or *OsABIL2*^*G183D*^ genes under the control of the *PGD1* promoter, a constitutively active promoter characterized in rice^36^. Each transgenic line possessed a single T-DNA in their genomes, and homozygous lines were established to evaluate ABA sensitivity (Supplementary Fig. S1).

**Figure 3:**
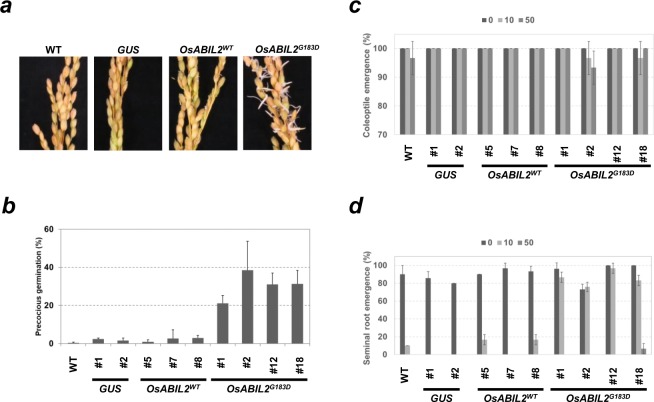
Dormancy and ABA sensitivity of seed from transgenic rice expressing *OsABIL2*^*WT*^ and *OsABIL2*^*G183D*^. (**a**) Precocious germination of panicles from WT, *GUS*, *OsABIL2*^*WT*^ and *OsABIL2*^*G183D*^ lines. Typical photographs were taken after precocious germination assay. (**b**) Precocious germination rates of WT, *GUS*, *OsABIL2*^*WT*^, and *OsABIL2*^*G183D*^ lines. Panicles from each genotype were harvested four weeks after ear emergence and used for precocious germination assay. Values with standard deviations are represented (n = 3). (**c**,**d**) The effects of exogenous ABA application on seed germination. The coleoptile and seminal root emergences from the hull were used as germination criteria. Twenty seeds of each line were used for the germination test. Each experiment was performed in triplicate. Standard deviations are represented as error bars (n = 3). Both rates were values at four days after starting germination at 28 °C.

It has been shown that seed dormancy is reduced in most ABA insensitive mutants, and the viviparous germination phenotype is obvious in both ABA deficient and insensitive mutants of cereals^37–39^. To examine the precocious germination rates, panicles setting premature seeds were harvested from wild-type rice (WT), *GUS*, *OsABIL2*^*WT*^ and *OsABIL2*^*G183D*^ lines. As shown in Fig. 3a, the emergence of the coleoptiles and the seminal roots were observed in panicles from transgenic lines expressing *OsABIL2*^*G183D*^, but not in the other genotypes. The precocious germination rates were approximately 20–40% in *OsABIL2*^*G183D*^ lines, and less than 3% in the other genotypes (Fig. 3b).

The germination rates of mature seeds were examined in the presence or absence of ABA. Rice germination began with the emergence of the coleoptile from then hull, and then the seminal root appeared^40^. The coleoptile emergence and seminal root emergence from the hull were used as germination criteria. Rates of coleoptile emergence were comparable among WT, *GUS*, *OsABIL2*^*WT*^, and *OsABIL2*^*G183D*^ lines with or without ABA (Fig. 3c). In contrast, differences were observed in the rates of seminal root emergence in the presence of ABA (Fig. 3d). *OsABIL2*^*G183D*^ lines showed more than 70% seminal root emergence, but the rates were 0–16% in WT, *GUS* and *OsABIL2*^*WT*^ lines with 10 μM ABA (Fig. 3d). Seminal root emergences of WT, *GUS*, *OsABIL2*^*WT*^, and *OsABIL2*^*G183D*^ lines were mostly suppressed with 50 μM ABA, while 6% emergence was observed in *OsABIL2*^*G183D*^ line #18-5 (Fig. 3d). These results suggested that the constitutive expression of *OsABIL2*^*G183D*^ weakened the seed dormancy of premature seeds, and reduced the ABA sensitivity of seminal root emergence during seed germination in rice.

### Overexpression of *OsABIL2*^*G183D*^ decreased ABA sensitivity in seedling

**Figure 4:**
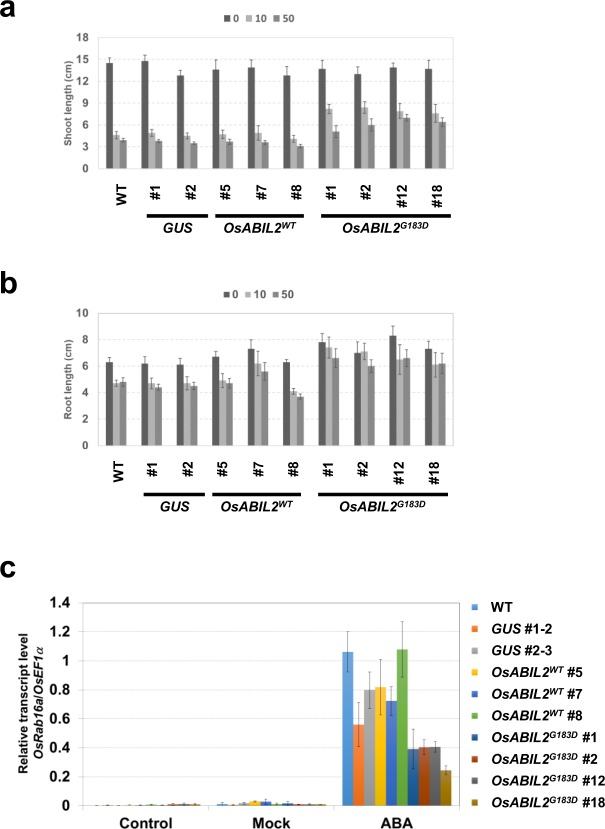
ABA responsiveness of transgenic rice seedling expressing *OsABIL2*^*WT*^ and *OsABIL2*^*G183D*^. (**a**,**b**) The effects of ABA on seedling growth. Seedlings of each transgenic line were grown onto liquid medium supplemented with 0, 10, and 50 μM ABA for seven days. The shoot and root lengths of each genotype grown without ABA were defined as 100%. The relative rates of the shoot or root lengths of each genotype grown with 10 or 50 μM ABA are shown. (**c**) Expression levels of *OsRab16b* transcription in response to ABA. Seedlings of each genotypes were subjected to 24 h of 10 μM ABA treatment. The gene expression of *OsRab16b* was analyzed using QRT-PCR. The expression levels of *OsRab16b* transcription were normalized with the *OsEF1α* transcription levels of corresponding samples. Error bars showed SD (n = 3).

To test the effect of ABA on seedling growth, WT and transgenic lines were grown in a hydroponic medium with or without ABA. The growths of both the shoot and root in all genotypes were inhibited by exogenous ABA (Fig. 4a,b). As compared to shoot lengths in the absence of ABA, 10 μM and 50 μM ABA decreased shoot lengths in WT, GUS, and *OsABIL2*^*WT*^ lines by 30–35% and 21–27%, respectively (Fig. 4a). When *OsABIL2*^*G183D*^ lines grew in media with 10 μM and 50 μM ABA, the rates of shoot lengths were reduced by 55–65% and 37–49%, respectively, relative to their shoot growths without ABA (Fig. 4a). A similar tendency was observed in the root growth with or without ABA. The relative growth rates of the root lengths of the NT, *GUS*, and *OsABIL2*^*WT*^ lines were reduced by 65–85% and 60–77% in the presence of 10 μM and 50 μM ABA, respectively (Fig. 4b). The relative root growth rates of *OsABIL2*^*G183D*^ lines were decreased by 78–100% and 80–85% with 10 μM and 50 μM ABA, respectively (Fig. 4b). The shoot and root growths of the *OsABIL2*^*G183D*^ lines were ABA insensitive as compared to those of the other genotypes (Fig. 4a,b). From these results, it was deduced that the ABA sensitivity of the *OsABIL2*^*WT*^ lines was mostly similar to that of the WT and *GUS* lines, whereas the *OsABIL2*^*G183D*^ lines were obviously insensitive to ABA during seedling growth.

Next, the gene expression of the ABA-inducible gene, *OsRab16b*, was examined after 24 h of 10 μM ABA treatment. The induction of *OsRab16b* transcription was observed in all genotypes. The levels of *OsRab16b* transcription differed between genotypes (Fig. 4c). The transcription levels of *OsRab16b* in *OsABIL2*^*G183D*^ lines were lower than those in the WT, *GUS*, and *OsABIL2*^*WT*^ lines (Fig. 4c). This result clearly indicated that ABA responsiveness was significantly reduced in the gene expression level of *OsABIL2*^*G183D*^ lines.

### Agricultural traits of ABA insensitive rice

**Figure 5:**
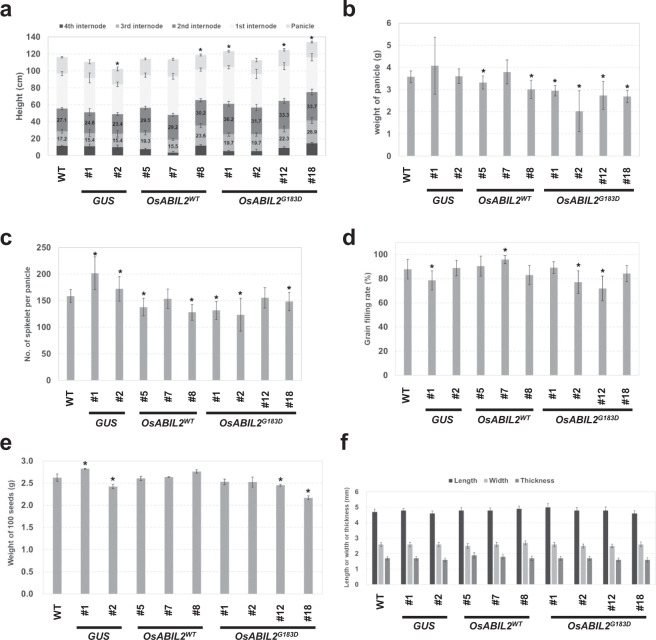
Agricultural traits of rice expressing OsABIL2^G183D^. (**a**) Plant heights consisted of the lengths of panicles and each internode. (**b**) Weight of panicle. (**c**) Grain numbers per panicle. (**d**) Grain filling rate. (**e**) 100-seed weight. (**f**) Length or width or thickness of rice seed. Asterisks above the bars indicate statistically significant differences between WT and transgenic lines (*t*-test, *P*-value < 0.05, n = 14–20, mean ± SD).

The various characters of the *OsABIL2*^*G183D*^ lines were investigated to address the effect of decreased ABA sensitivity on agricultural traits. The lines were grown in soil in a greenhouse until mature grains were produced. Significant differences were observed in plant height, weight of panicle, and number of spikelets per panicle. Three of four *OsABIL2*^*G183D*^ lines grew taller than the WT (Fig. 5a). A clear difference was seen in the second and third internodes. The respective lengths of the second and third internodes in the all *OsABIL2*^*G183D*^ lines were 116–133% and 114–156% compared to those in the WT (Fig. 5a). The weight of panicles was decreased to 84–92% in two of three *OsABIL2*^*WT*^ lines, and 56–83% in all *OsABIL2*^*G183D*^ lines, relative to that in WT (Fig. 5b). Spikelet numbers in the *GUS* line were increased by 108–127% (Fig. 5c), whereas they were reduced by 80–87% in two of three *OsABIL2*^*WT*^ lines, and by 77–94% in all *OsABIL2*^*G183D*^ lines, compared to that in the WT (Fig. 5b). Significant were observed in each transgenic line for variables such as the rate of grain filling (Fig. 5d), weight of grains (Fig. 5e), and grain size (Fig. 5f), but these differences were not consistent among each genotype. Therefore, we concluded that there were no obvious effects of decreased ABA sensitivity on the rates of those three traits (Fig. 5d–f). Therefore, the decreased panicle weights in *OsABIL2*^*G183D*^ lines were caused by the reduced number of spikelets per panicle (Fig. 5c) but not by the other factors in the panicle (Fig. 5d–f).

### Plant growth of *OsABIL2*^*G183D*^ lines was improved under low temperature conditions in rice

**Figure 6:**
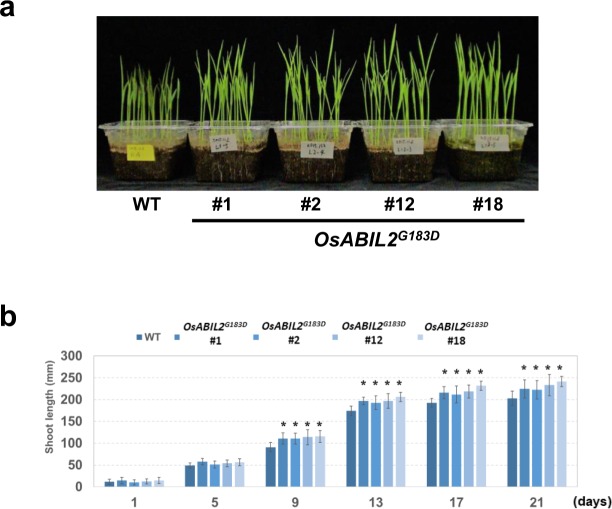
Growth of transgenic rice expressing *OsABIL2*^G183D^ under low temperature conditions. (**a**) Photograph of WT and transgenic lines grown at 15 °C. Seeds of each genotype were soaked in water for three days at 28 °C in the dark. After soaking, 20 germinated seeds each of genotypes were planted in a plastic container with culture soil. Plants were grown in at 15 °C for 21 days. The photograph was taken after 21 days of 15 °C incubation. (**b**) Growth of transgenic rice expressing *OsABIL2*^G183D^ at 15 °C. Shoot lengths of each genotype were measured at intervals of four days for 21 days. Error bars indicate SD (n = 20). Asterisks above the bars present statistically significant differences between WT and transgenic lines (*t*-test, *P*-value < 0.05, n = 14–20, mean ± SD).

The decreased levels of ABA in rice have previously been shown to improve seedling vigor under low temperatures^21^. To evaluate the seedling vigor of *OsABIL2*^*G183D*^ lines under low temperature conditions, WT and *OsABIL2*^*G183D*^ lines were grown at 15 °C, and their growth was monitored for 21 days. *GUS* and *OsABIL2*^*WT*^ lines were excluded from this experiment since their ABA sensitivity was comparative to that of WT. After nine days of growth at 15 °C, the heights of *OsABIL2*^*G183D*^ transgenic plants were higher than those of WT plants (Fig. 6a). As shown in Fig. 6b, *OsABIL2*^*G183D*^ lines grew faster than the WT plants. This result indicated that decreased ABA sensitivity improved seedling vigor under low temperature conditions.

## Discussion

In this study, we generated transgenic *Arabidopsis* and rice expressing *OsABIL2*^*WT*^ or *OsABIL2*^*G183D*^, and the ABA sensitivity of these transgenic lines was comparatively investigated. Although it has been reported that the transgenic rice expressing *OsABIL2*^*WT*^ presented ABA insensitivity and morphological changes in the root^28^, we utilized both *OsABIL2*^*WT*^ and *OsABIL2*^*G183D*^ to evaluate the effect of *abi1-1* type mutation in OsABIL2 protein on ABA sensitivity in plants. As a result, the transgenic *Arabidopsis* and rice expressing *OsABIL2*^*G183D*^ represented stronger ABA insensitivities than did the transgenic plants that expressed *OsABIL2*^*WT*^.

Dimeric ABA receptors interacted with clade A PP2Cs in an ABA-dependent manner^18,41^. OsABIL2 exhibited ABA-dependent interactions with OsPYL1 (Fig. 1). In addition, OsABIL2^G183D^ did not interact with OsPYL1 in the presence or absence of ABA (Fig. 1). The interactions of ABI1, ABI2, and HAB1 proteins with the ABA receptors were abolished by introduction of the *abi1-1* type mutation into them^42,43^. The ectopic expression of *OsABIL2*^*G183D*^ led to an obvious ABA-insensitive phenotype in *Arabidopsis* and rice (Figs 2–4). The transgenic plants that expressed *OsABIL2*^*G183D*^ showed stronger ABA insensitivity than transgenic plants expressing *OsABIL2*^*WT*^ (Figs 2–4). Umezawa *et al*., reported that *Arabidopsis* PP2C harboring the *abi1-1* type mutation did not show an ABA-dependent interaction with dimeric ABA receptors, but did with SnRK2^42^. The ABA receptor-insensitive PP2C could constantly bind to SnRK2 via each catalytic cleft and inhibit the activity of SnRK2^11^. The binding affinity of the abi1-1 protein against SnRK2 may be higher than that of ABI1 against SnRK2^42^. This property of the abi1-1 protein led to the ABA-insensitive phenotype. This may be because the constitutive expression of *OsABIL2*^*G183D*^ brings stronger ABA insensitivity than that of *OsABIL2*^*WT*^ in *Arabidopsis* and rice.

The ABA sensitivity of seed germination was affected by the ectopic expression of *OsABIL2*^*G183D*^ in *Arabidopsis* and rice. Visible changes in the seed germination of *Arabidopsis* and cress first occurred during testa rapture, and the cotyledon then started to develop after radicle emergence^44^. In these plants, exogenous ABA is thought to affect germination after testa rapture^44^. As shown in Fig. 2c,d, exogenous application of ABA delayed the germination processes in non-transgenic *Arabidopsis*, however *abi1-1* and transgenic *Arabidopsis* expressing *OsABIL2*^*G183D*^ represent an ABA resistant germination phenotype. In case of rice seed germination, coleoptile emergence occurs first, followed by the seminal root emerged from hull^40^. Although coleoptile emergence was not suppressed by 50 μM ABA in any of the trialed genotypes (Fig. 3c), 10–50 μM ABA strongly inhibited seminal root emergence in WT, *GUS*, and *OsABIL2*^*WT*^ lines other than *OsABIL2*^*G183D*^ lines (Fig. 3d). In *Arabidopsis*, the inhibition of cotyledon greening by exogenous ABA was stronger than that of radicle emergence (Fig. 2c,d). In rice, the application of ABA suppressed seminal root emergence, but not coleoptile emergence (Fig. 3c,d). In both plant species, later processes were highly inhibited by ABA. Therefore, the degree of inhibition by exogenous ABA might be dependent on the order in which the processes occur, or on the differences in ABA sensitivity between the shoot and root. Until now, mutants with a defect in ABA biosynthesis or sensitivity have been isolated by the mutant screening of viviparous germination or ethylene sensitivity in rice^39,45^. To identify ABA insensitive mutants in rice more effectively, the ABA sensitivity of rice seed could be clearly distinguished by seminal root emergence.

Li *et al*.^28^ reported that rice seedlings expressing *OsABIL2*^*WT*^ showed strong ABA insensitivity, such as the wilty and seedling lethal phenotypes. On the other hand, transgenic rice expressing *OsABIL2*^*G183D*^ showed mild ABA insensitivity, and *OsABIL2*^*WT*^ lines showed similar ABA sensitivity to WT in rice (Fig. 4a,b). The difference in ABA insensitivity observed between our study and the previous report could be because of a number of reasons. One reason could be that the difference in promoter that regulates the expression levels of the *OsABIL2* gene; the *PGD1* promoter was used in our study, while Li *et al*.^28^ used the 35S promoter. Since guard cells largely control the transpiration of water in plants, the activity of the *PGD1* promoter in them may be lower than that of the 35S promoter. Another reason may be the difference in translationally fused tags to OsABIL2. We fused GFP to the amino-terminal of OsABIL2, and Li *et al*.^28^ attached a FLAG tag to carboxyl-terminal of OsABIL2. Although it was shown that transgenic *Arabidopsis* expressing GFP-abi1-1 or abi1-1 proteins presented similar insensitivity to ABA^46^, translationally fused GFP might reduce the inhibitory function of OsABIL2^G183D^ against SnRK2 orthologs in rice.

Since we previously demonstrated that decreased levels of endogenous ABA improved the growth of rice seedlings under low temperatures^21^, we thought that reduced ABA sensitivity would also have a beneficial impact on seedling vigor. As hypothesized, ABA-insensitive rice expressing *OsABIL2*^*G183D*^ showed improved growth at 15 °C (Fig. 6a,b). On the other hand, constitutive expression of *OsABIL2*^*G183D*^ led to elongated internodes (Fig. 5a). Similar tendencies were observed in the ABA deficient mutant *mhz4* defect in neoxanthin synthase^45^. In contrast, the overexpression of *OsPYL5* increased ABA sensitivity, and resulted in shorter internode lengths^47^. ABA is well known to act antagonistically against gibberellin (GA) in various plant species^48–50^. Internode elongation is mainly controlled by GA in rice^51,52^. The internode elongation noted in *OsABIL2*^*G183D*^ lines and the *mhz4* mutant might be caused by the augmented sensitivity to GA resulting from an attenuated ABA signal. In relation to the effect of GA, seedling vigor was ameliorated by increased endogenous GA levels during seedling growth^53^. The rice cultivar ‘Dunghan Shali’ showed rapid growth, and a QTL analysis revealed that this trait was closely related to polymorphisms in the promoter region of *OsGA20ox1* encoding the GA biosynthetic enzyme. The levels of *OsGA20ox1* transcript and GA present in ‘Dunghan Shali’ seedlings were significantly higher than those in other parental Japonica varieties^53^. Taken together, the improvements in seedling growth under low temperature conditions may be because of the effect of increased GA signaling arising from decreased ABA sensitivity.

This study demonstrated that ABA insensitive transgenic rice expressing *OsABIL2*^*G183D*^ represented improved rice seedling vigor under low temperatures, as well as agriculturally negative traits such as precocious germination, elongated internodes, and reduced yields (Figs 5 and 6). We previously reported that ABA-deficient rice generated by the constitutive expression of the ABA-inactivating enzyme demonstrated not only low temperature resistant growth, but also drought hypersensitive phenotypes^21^. Although it is possible that a potentially ABA-insensitive variety may not be preserved during rice breeding because of undesired traits, pollen-specific reductions in ABA levels in response to drought or cold stress can bring resistance to stress-induced pollen sterility^20^. Therefore, the reduction in ABA sensitivity should be controlled not in a constitutive manner, but in a tissue-specific or stress-inducible manner. The application of a low temperature-inducible promoter to drive *OsABIL2*^*G183D*^ could enhance plant performance only under low temperature conditions, without the negative traits resulting from constitutive ABA insensitivity. The next challenge will be to tune ABA sensitivity to improve plant growth in response to various environmental changes.

## Methods

### Plant materials

*Arabidopsis thaliana* (Columbia accession) and the *Oryza sativa* Japonica rice cultivar ‘Kitaaoba’ were used in this study. Plant growth conditions were as described later in this section.

### Cloning and PCR mutagenesis of *OsABIL2*

*OsABIL2* ORF was amplified from first strand cDNA by using PrimeSTAR GXL DNA polymerase (TaKaRa, Shiga, Japan). The ORF was introduced into pENTR D/TOPO vector. To generate mutant-type OsABIL2 harboring an equivalent mutation to *abi1-1*, the 183rd glycine (G) of OsABIL2 was replaced with aspartic acid (D) using a PrimeSTAR PCR mutagenesis kit (TaKaRa, Shiga, Japan). The primers used in these experiments are listed in Supplementary Table S1.

### Yeast two-hybrid assay

A gateway cassette was amplified from pMDC43 vector by PCR using PrimeSTAR GXL DNA polymerase (TaKaRa, Shiga, Japan). The cassette was cloned into the multicloning site (between *Eco*R I and *Bam*H I) of pGADT7. *OsABIL2*^*WT*^ and *OsABIL2*^*G183D*^ were transferred from cloning vectors to the gateway cassette in pGADT7 to generate prey vectors. *OsPYL1* ORF was amplified from first strand cDNA and cloned into a multicloning site (between *Eco*R I and *Bam*H I) of pGBKT7 vector using an Infusion HD cloning kit (Clontech). Each prey vector was introduced into the yeast strain Y2H Gold with the bait vector, *OsPYL1* pGBKT7. Primers used in these experiments are listed in Supplementary Table S1. Transformed yeast cells were incubated on the double dropout medium (Leu and Trp were omitted from the medium) to screen cells harboring both bait and prey vectors. To confirm the effect of ABA on the interaction between OsABIL2 and OsPYL1, transformed cells were incubated on the quadruple dropout medium (Leu, Trp, Ade, and His were excluded from the medium) supplemented with or without 10 μM ABA.

### Generation of transgenic *Arabidopsis*

To construct the binary vector harboring *OsABIL2*^*WT*^ and *OsABIL2*^*G183D*^, *OsABIL2*^*WT*^, and *OsABIL2*^*G183D*^ were transferred from the cloning vectors to the pEarleyGate201 vector using LR clonase (Life Technologies). The resultant binary vectors were introduced into Agrobacterium GV3101. *Arabidopsis* transformation was performed by the floral dip method. Transgenic *Arabidopsis* harboring a single copy of the T-DNA insertion was screened onto MS medium (5 mM MES-KOH pH 5.8, 1/2 strength MS salt mixture, 0.5% sucrose, 0.5% gellan gum) containing 10 mg/L of glufosinate ammonium (Wako, Japan), and homozygous lines were used for the series of experiments.

### Germination assay of *Arabidopsis* seeds

*Arabidopsis* seeds of Col, *OsABIL2*^*WT*^, and *OsABIL2*^*G183D*^ lines were surface-sterilized and sown onto MS medium containing several concentrations of ABA. After two days of stratification at 4 °C in darkness, plates were incubated at 22 °C under continuous light for seven days, and the rates of germination or seedling establishment were counted daily. Germination was scored based on radicle emergence, whereas seedling establishment was measured based on the greening of cotyledons.

### Water loss assay of *Arabidopsis*

*Arabidopsis* seeds were grown on the MS medium for seven days as described above. Seven-day-old plants were transferred to soil. After the plants had grown on soil for four weeks, their shoots were removed and placed in a plastic tray for 3 h to dehydrate. Their decreasing weights were measured at 0, 30, 90, 150, and 210 min after detachment.

### Generation of transgenic rice

To generate overexpression constructs, a 35S promoter within the pMDC43 vector was replaced with a PGD1 promoter. First, the pMDC43 vector was digested by the restriction enzymes *Kpn* I and *Hin*d III to remove the 35S promoter region. Then, 1891 bp of the *PGD1* promoter region, including the first intron, was amplified by PCR following the methods of Park *et al*.^36^. The fragment of the *PGD1* promoter was introduced into the digested site of pMDC43 using an Infusion HD cloning kit (Clontech). DNA fragments of *OsABIL2*^*WT*^, *OsABIL2*^*G183D*^, or *GUS* were transferred from the cloning vectors to the *PGD1* promoter pMDC43 vector using LR clonase (Life Technologies). Agrobacterium (EHA105) was transformed with the binary vectors and used for rice transformation according to the methods of the previous report^54^. Transgenic rice harboring one or two copies of T-DNA insertion was selected by Southern blotting. The homozygous T3 generation was used in this study.

### Germination assay of rice

For the germination assay, Kitaaoba or independent transgenic plants were grown in a greenhouse over the same period. The greenhouse conditions were as follows: 16 h of day at 25 °C and 8 h of night at 20 °C, with 50–70% humidity. The precocious germination assay was performed following the instructions from the previous report^55^. Briefly, the panicles were harvested at four, five, and six weeks after ear emergence. Harvested panicles were incubated on wet paper towels for seven days at 30 °C in the dark, and germination rates were counted. To examine the germination rates of mature seeds, the harvested seeds were dehydrated in an incubator set at 30 °C for one week. Twenty dry seeds from each genotype were washed with sterilized water. Then, each batch was incubated in 20 mL of sterilized water in plastic Petri dishes. The Petri dishes were incubated at 28 °C in the dark, and the germination rate was measured daily.

### The effects of ABA on growth of rice seedlings or gene expressions

Seed surfaces were sterilized, and sterilized seeds were germinated in the dark at 28 °C for three days. Germinated seeds were hydroponically cultured in the plant growth chamber for four days under a long-day condition (light for 16 h at 25 °C and dark for 8 h at 20 °C, light intensity: ~110 μmol m^−2^ s^−1^), and then plants were grown for an additional seven days on a hydroponic culture medium containing 10 mM MES-KOH pH 5.8, with or without each concentrations of ABA (10 or 50 μM). After the ABA treatments, the shoot and root lengths were measured. The dry weights of plantlets were determined after three days of dehydration at 70 °C. For gene expression analysis, plants were treated with 10 or 50 μM ABA for 24 h. ABA-treated samples were frozen with liquid nitrogen and stored at −80 °C until use.

### RNA extraction and quantitative reverse transcription (QRT)-PCR

Frozen samples were homogenized using the Multi-beads Shocker (Yasuikikai). An RNeasy mini kit (Qiagen) was used for RNA extraction. RNA samples were treated with DNaseI RNase-free TaKaRa, Shiga, Japan), and subjected to phenol–chloroform extraction and LiCl precipitation. The first strand cDNA was synthesized from 500 ng of total RNA by using PrimeScript RT Master Mix (TaKaRa, Shiga, Japan). The LightCycler 96 system (Roche) was used for QRT-PCR with FastStart essential DNA Probes Master (Roche) and TaqMan probe (Roche). *Elongation factor 1α* (*EF1α*) gene was used as an internal standard gene. The primer sequences with a TaqMan probe are listed in Supplementary Table S2.

### Low temperature growth of transgenic rice expressing *OsABIL2*^*G183D*^

The surface-sterilized seeds of each genotype were washed with sterilized water and soaked in water for three days at 28 °C in the dark. After soaking, 20 germinated seeds of each genotype were planted in a 7 cm square plastic container with 180 g of culture soil. Plants were grown in a chamber (12 h light, 12 h dark, 15 °C, light intensity: 160 μmol m^−2^ s^−1^) for 21 days. Shoot lengths of each genotype were measured at 1, 5, 9, 13, 17, and 21 days after planting.

## Electronic supplementary material

Supplementary Information
