# Supplementary material for: Ectopic expression of mutated type 2C protein phosphatase OsABI-LIKE2 decreases abscisic acid sensitivity in Arabidopsis and rice
Source: Sci Rep. 2018 Aug 17;8:12320. doi: 10.1038/s41598-018-30866-z (PMC6097999; doi:10.1038/s41598-018-30866-z)
Supplement: Supplementary file 1 — Supplementary Information [file 41598_2018_30866_MOESM1_ESM.pdf]

Supplementary Information

Ectopic expression of mutated type 2C protein phosphatase, OsABIL2 decreases abscisic acid sensitivity in Arabidopsis and rice

Author

Akira Endo[a, 1], Chika Egawa[a, 2], Mihoko Oohashi[a, 3], Ayano Meguro-Maoka[a], Etsuo Shimosaka[a] and Yutaka Sato[a, b, †]

Affiliation

[a] Hokkaido Agricultural Research Center, National Agriculture and Food Research Organization (NARO), Toyohira, Sapporo 062–8555, Japan

[b] Division of Bio-systems Sustainability, Graduate School of Agriculture, Hokkaido University, N9 E9, Kita-ku, Sapporo 060-8589, Japan

[1]Present address: Plant Genome Engineering Research Unit, Institute of Agrobiological Sciences, NARO, 2-1-2 Kannondai, Tsukuba 305-8602, Japan

[2]Present address: Institute for Agro-Environmental Sciences, NARO, 3-1-3 Kannondai, Tsukuba 305-8604, Japan

[3]Present address: Biomedical research, Education instrumentation center, Sapporo Medical University, S1 W17, Chuo-ku, Sapporo 060-8556, Japan

[†]**Corresponding author**: Dr. Yutaka Sato

E-mail: yutaka@affrc.go.jp

Running title: Improvement of plant growth under low temperature by decreasing ABA sensitivity

Key words: rice / abscisic acid / low temperature /seedling vigor

Figure S1 Southern blot analysis of genomic DNA samples from transgenic rice plants using GFP probe.

Each blot was hybridized with alkaline phosphatase-labeled GFP probe and each lane contains approximately 10 μg of EcoRI-digested genomic DNA isolated from leaf tissue. Genotypes and line numbers were indicated above each gel image. W: genomic DNA from wild-type plants, V: vector DNA that introduced into the GFP-GUS transgenic lines.

Table S1. Primers used for cloning and vector construction.

Primers used in the cloning and PCR mutagenesis of OsPP2C53	
Primer name	Primer sequence (5' to 3')
OsPP2C53 cDNA F	CTGTGCTGGATCGAGTCGAG
OsPP2C53 cDNA R	CTGCAGCAGCACCAGTACTG
OsPP2C53 pENTR F	CACCATGGAGGACCTCGCCCTGCCC
OsPP2C53 pENTR R	TCATGCTTTGCTCTTGAACTTCCT
OsPP2C53 G183D F	GGCCACGATGGCGTTCAGGTTGCCAAT
OsPP2C53 G183D R	AACGCCATCGTGGCCATCGTAGACGGC
Primers used for yeast two hybrid assay	
Primer name	Primer sequence (5' to 3')
GW F	agctatcaaacaagtttgtac
GW R	tatcgaaccactttgtacaag
GW IF pGADT7 F	GGAGGCCAGTGAATTCAGCTATCAAACAAGTTTGTAC
GW IF pGADT7 R	CGAGCTCGATGGATCCTATCGAACCACTTTGTACAAG
OsPYL1 cDNA F	CATCTGGCTCGCTCGACATC
OsPYL1 cDNA R	GCAGCACCACCAGGAATGAG
OsPYL1 IF pGBKT7 F	CATGGAGGCCGAATTCATGGAGCAGCAGGAGGAAGTGCCA
OsPYL1 IF pGBKT7 R	GCAGGTCGACGGATCCCTATTCCGCCGCCGCCGGTGGAGG
Primers used to construct the binary vector	
Primer name	Primer sequence (5' to 3')
PGD1pro F	TAGATATGCCGAACATGACC
PGD1pro R	GCAGATAGATGCACCAAATG
PGD1 IF F	GGCCAGTGCCAAGCTTTAGATATGCCGAACATGACC
PGD1 IF R	TCATTTTTTCTACCGGTACCGCAGATAGATGCACCAAATG

Table S2. Primers and TaqMan probes used in QRT-PCR

Gene Name	Gene ID	TaqMan Probe	Primer Set (5' to 3')	
	RAP-DB		Forward Primer	Reverse Primer
OsRab16A	Os11g0454300	#78	gagggaggagcacaagacc	attccatcatcctcagacgag
EF1a	Os03g0177500	#55	cctcctcctctcgccatc	ttctccttacccatggttgatt

Figure S1

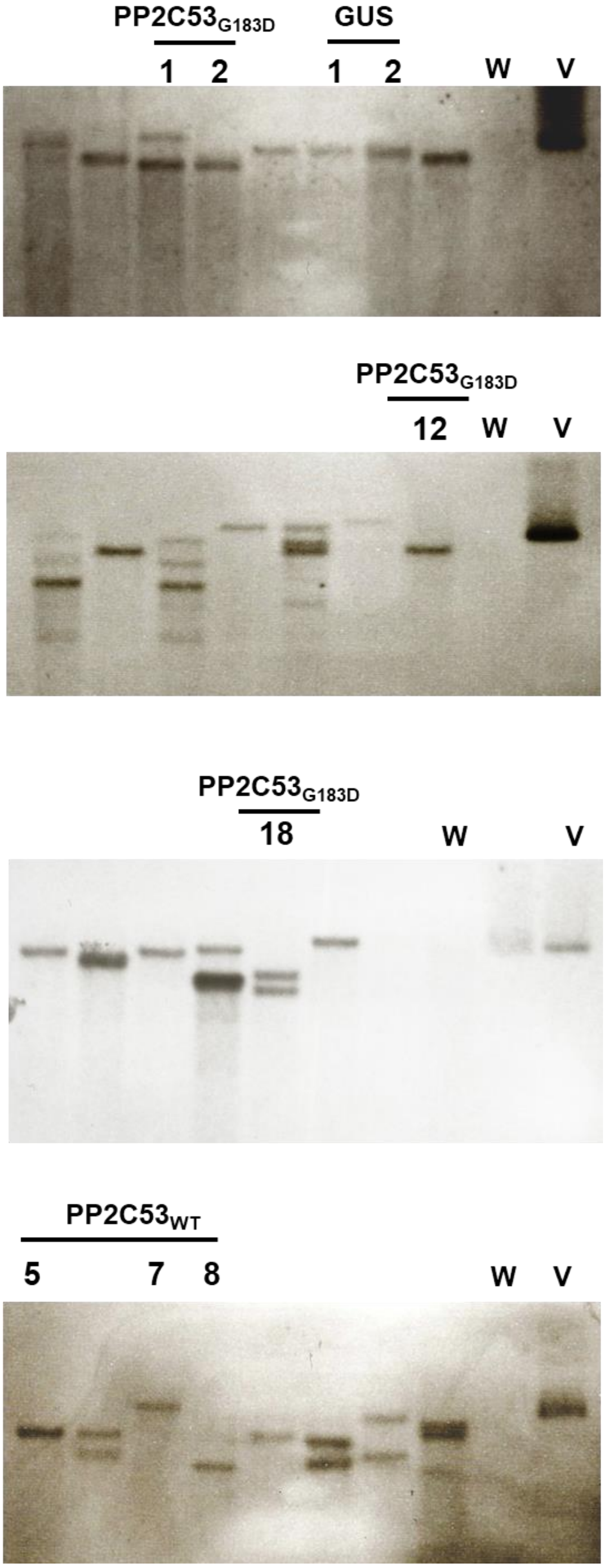
PP2C53G183D
GUS
1 2 1 2 W V
PP2C53G183D
12 W V
PP2C53G183D
18
W V
PP2C53WT
5 7 8
W V
